# Effect of seasonal malaria chemoprevention plus azithromycin on *Plasmodium falciparum* transmission: gametocyte infectivity and mosquito fitness

**DOI:** 10.1186/s12936-021-03855-3

**Published:** 2021-07-27

**Authors:** Koudraogo Bienvenue Yaméogo, Rakiswendé Serge Yerbanga, Seydou Bienvenu Ouattara, Franck A. Yao, Thierry Lefèvre, Issaka Zongo, Frederic Nikièma, Yves Daniel Compaoré, Halidou Tinto, Daniel Chandramohan, Brian Greenwood, Adrien M. G. Belem, Anna Cohuet, Jean Bosco Ouédraogo

**Affiliations:** 1 Institut de Recherche en Sciences de la Santé, Bobo-Dioulasso, Burkina Faso; 2 Université Nazi Boni, Bobo-Dioulasso, Burkina Faso; 3 Institut des Sciences et Techniques (INSTech Bobo), BP2779 Bobo-Dioulasso, Burkina Faso; 4 MIVEGEC, University of Montpellier, IRD, CNRS, Montpellier, France; 5 Laboratoire Mixte International Sur Les Vecteurs (LAMIVECT), Bobo Dioulasso, Burkina Faso; 6 Centre de Recherche en Écologie et Évolution de la Santé (CREES), Montpellier, France; 7 Institut de Recherche en Sciences de la Santé, Nanoro, Burkina Faso; 8 London School of Hygiene and Tropical Medicine, London, UK

**Keywords:** Seasonal malaria chemoprevention, Azithromycin, Gametocytes, Transmission

## Abstract

**Background:**

Seasonal malaria chemoprevention (SMC) consists of administration of sulfadoxine-pyrimethamine (SP) + amodiaquine (AQ) at monthly intervals to children during the malaria transmission period. Whether the addition of azithromycin (AZ) to SMC could potentiate the benefit of the intervention was tested through a double-blind, randomized, placebo-controlled trial. The effect of SMC and the addition of AZ, on malaria transmission and on the life history traits of *Anopheles gambiae* mosquitoes have been investigated.

**Methods:**

The study included 438 children randomly selected from among participants in the SMC + AZ trial and 198 children from the same area who did not receive chemoprevention. For each participant in the SMC + AZ trial, blood was collected 14 to 21 days post treatment, examined for the presence of malaria sexual and asexual stages and provided as a blood meal to *An. gambiae* females using a direct membrane-feeding assay.

**Results:**

The SMC treatment, with or without AZ, significantly reduced the prevalence of asexual *Plasmodium falciparum* (LRT X^2^_2_ = 69, P < 0.0001) and the gametocyte prevalence (LRT X^2^_2_ = 54, P < 0.0001). In addition, the proportion of infectious feeds (LRT X^2^_2_ = 61, P < 0.0001) and the prevalence of oocysts among exposed mosquitoes (LRT *X*^*2*^_2_ = 22.8, P < 0.001) was reduced when mosquitoes were fed on blood from treated children compared to untreated controls. The addition of AZ to SPAQ was associated with an increased proportion of infectious feeds (LRT X^2^_1_ = 5.2, P = 0.02), suggesting a significant effect of AZ on gametocyte infectivity. There was a slight negative effect of SPAQ and SPAQ + AZ on mosquito survival compared to mosquitoes fed with blood from control children (LRTX^2^_2_ = 330, P < 0.0001).

**Conclusion:**

This study demonstrates that SMC may contribute to a reduction in human to mosquito transmission of *P. falciparum*, and the reduced mosquito longevity observed for females fed on treated blood may increase the benefit of this intervention in control of malaria. The addition of AZ to SPAQ in SMC appeared to enhance the infectivity of gametocytes providing further evidence that this combination is not an appropriate intervention.

## Background

Malaria remains a major cause of morbidity and mortality, particularly in Africa where 94% of cases occur. Effective management of clinical cases associated with the use of insecticide-impregnated material has been effective in reducing malaria transmission and has greatly contributed to the progresses in the fight against the disease since the beginning of the century. However, the reduction of malaria burden has stalled, or even reversed, in some countries in the recent years and additional strategies are needed. These include chemopreventive strategies such as intermittent preventive treatment during pregnancy (IPTp) and seasonal malaria chemoprevention (SMC) for children [1]. SMC consists of administration of therapeutic doses of sulfadoxine-pyrimethamine (SP) + amodiaquine (AQ) at monthly intervals to children aged 3–59 months during the peak malaria transmission period in areas where malaria transmission is seasonal [2]. In 2019, 21.5 million children in 13 countries in Africa’s Sahel and sub-Sahel sub-region received malaria prophylaxis through SMC programmes [1]. SMC proved to be safe and cost-effective when deployed at the population level [3]. Other advantages of SMC are that it relies on frequent interactions between health staff or training volunteers and young children, which provides an opportunity for further benefits for populations, such as preventive administration of other drugs [4]. Mass administration of azithromycin (AZ) has been reported to provide a reduction in all cause mortality [5, 6]. Therefore, to determine whether the addition of AZ to SMC could reduce child mortality and morbidity was tested through a double-blind, randomized, placebo-controlled trial in young children in Burkina Faso and Mali. This trial showed that the addition of AZ reduced the incidence of several infectious diseases but did not reduce the incidence of deaths or hospital admissions [4]. Addition of AZ to SMC had little effect on the incidence of clinical attacks of malaria, but it may have had some effect on malaria transmission, and determining whether this was the case warrants further attention.

The transmission of malaria parasites from human to mosquito vector requires the presence of mature *Plasmodium* gametocytes in the ingested blood and both host and parasite factors can influence the production of gametocytes and their infectivity to mosquitoes [7, 8]. A large panel of drugs, including anti-malarial molecules, show positive or negative influence on gametocytogenesis, on gametocyte infectivity, and/or on parasite development in the mosquito [9–11] and SMC, with or without additional drugs, is therefore expected to have an effect on human to mosquito transmission.

SP, and AQ to a lesser extent, have been shown to enhance the number of gametocytes circulating in the peripheral blood [12–14], possibly due to the release of sequestered parasites or through natural periodicity of parasite development [15]. However, the infectivity of gametocytes following SP treatment was low [16, 17]. In addition, SP has been shown to have a deleterious effect on mosquito survival [17], reflecting the complex effects of drug administration on malaria transmission. Mass administration of antibiotics to humans may affect the mosquito microbiota and possibly influence malaria parasite transmission in this way. Indeed, gut-inhabiting bacteria have been shown to interfere with parasite transmission in the mosquito and to modulate vector competence [18–24]. For instance, the presence of *Enterobacteriaceae,* such as *Serratia marcescens,* reduced the prevalence and intensity of infection [20] or even conferred refractoriness [20, 21, 24–26]. Experimental administration of antibiotics to mosquitoes either facilitated or impeded their infection, which suggested that antibiotics circulating in the blood of human hosts may impact the susceptibility of blood-sucking *Anopheles gambiae* females to transmit malaria infection by disturbing their gut microbiota [27]. Moreover, other parameters influencing vector capacity, such as mosquito lifespan and fecundity, are also affected by ingestion of antibiotics [28]. AZ reduced the *Plasmodium falciparum* gametocyte exflagellation process [29] and negatively affected the *P. falciparum* infection load as well as the mosquito lifespan [28] suggesting a potential for AZ to reducing malaria transmission.

The present study aimed at evaluating the effect of SMC with or without the addition of AZ on malaria transmission. It relied on a clinical trial that evaluated the benefit of adding AZ to SPAQ [4] and assessed the effect of SPAQ and SPAQ + AZ on the transmissibility of *P. falciparum* and on the life history traits of *An. gambiae* mosquitoes.

## Methods

### Study site

The study was conducted in Houndé health district, located about 100 km from Bobo-Dioulasso along the paved road to Ouagadougou, Burkina Faso. In this area, the rainy season lasts approximately six months from May to October. The main malaria vectors are reported to be *Anopheles gambiae, Anopheles coluzzii* [30]; *Anopheles arabiensis* is a secondary vector in the area [31]. *Plasmodium falciparum* is the most prevalent malaria parasite [32].

Child mortality caused by malaria remains high in Houndé in 2013 (36–40%) [33]. Malaria transmission occurs during and shortly after the rainy season. Treatment based on artemisinin combination therapy (ACT) and bed-net coverage are major malaria control interventions prior to the study. SMC or mass administration of AZ was not deployed at this site prior the study, but now the site areas receive SMC provided by the national malaria control programmes [4].

In the SMC + AZ trial, the percentage of children who received at least three directly observed cycles of the assigned regimen was 86.8 and 84.3%, respectively in 2015 and 2016 [4].

The population was estimated at 329,162 inhabitants in the Houndé health district in 2019 and is divided into villages over an area of 5622 sq km [34, 35]. Four villages: Koumbia, Dougmato, Kari and Boni, among the sites included in the SMC + AZ trial were selected randomly for this study.

Four month, each year, during the peak malaria transmission, study participants received SMC during 3 days. In addition, they received AZ or matching AZ placebo. All treatment doses were based on age and administered by study staff. The village of Pè in the district of Houndé, where SMC was not yet in place, was selected as a control.

### Study participants

The study participants were age 24–59 months at the start of the study. For ethical consideration, subjects aged 3–24 months from whom venous blood collection may often present difficulties did not select. Sample size was estimated on the basis of calculations for comparing SMC alone *versus* SMC + AZ, and SMC versus no SMC. Based on the weekly survey during the first year of the study, assuming that with SMC to have about 5% of mosquitoes infected, and in control children, about 15% or more of mosquitoes infected; if AZ reduces mosquito infectivity by 50%, to have 80% power to detect a difference, in total at least 20 to 30 children in each group, with 50 mosquitoes fed on each child would be needed.

In the four treated villages involved in the SMC + AZ trial [4], 438 participants were randomly selected from among the 21,737 participants for inclusion in the transmission study. The inclusion criteria were age 24–59 months and parental or other legal guardian consent. Exclusion criteria were chronic illness, symptomatic malaria, planned long-term absence from the village during the study period, or absence at the moment of inclusion, difficulty in collecting venous blood, or retraction of consent. In the control group village of Pê, 198 participants were selected according to the same inclusion/exclusion enrolment criteria used in the villages where chemoprevention was provided. Information about the trial recruitment was provided during meetings held in the medical centre. Ethical approval for the study was obtained from the national ethic committee of Burkina Faso under the Registration No. 2015-5-56.

### Treatment

For the participants who were part of the SMC + AZ trial, anti-malarials were administered by trial staff monthly between August and November according to the agenda of the trial and to be in the line with the annual peak of malaria transmission season [4].

Children received 500 mg of SP and 25 mg of pyrimethamine plus 150 mg of AQ on day 1 and 150 mg of AQ on days 2 and 3 (Guilin Pharmaceutical, Shanghai, China). In addition, they were assigned to receive either 200 mg of AZ or matching placebo on days 1, 2, and 3 (Cipla, Mumbai, India) through double-blinded administration by trial staff [4]. In the village Pê, children did not receive prophylactic anti-malarials. For all participants, an artemether–lumefantrine treatment was provided when malaria was diagnosed.

### Blood collection

At the end of each of the eight rounds of drug administration sessions (four sessions per year for two years) and in each of the four villages included in the SMC + AZ trial, 12 to 18 children were randomly selected for blood collection between 14 and 21 days after the start of the administration of prophylactic drugs. Evaluating the effect of chemoprophylaxis on man-to-mosquito transmission in the time frame of two to three weeks after the treatment, while children receive a treatment every four weeks, is a reflection of the effect of drug administration over the whole treatment season. In parallel to the eight treatment sessions, at least 25 untreated children of the control village were randomly selected from census list. For each selected child, 3 ml of blood were collected into a heparinized tube and kept at 37 ℃ in an incubator (ENKAB MODEL 70) for transportation to the laboratory in Bobo Dioulasso for 2–3 h before mosquito feeding. On arrival in the laboratory, thick blood smears were realized using 5 µl of blood.

### Microscopy

Thick blood smears were stained with Giemsa **(**Quimica Clinica Aplicada 990939**)** and independently examined by three experienced microscopists. Gametocytaemia was based on a count of the number of gametocytes per 1000 leukocytes, and asexual parasitaemia on the count of trophozoites and schizonts against 500 leukocytes, assuming an average number of 8000 leukocytes per µl of blood.

Criteria for concordance and determination of a final result were that after the three reports, results were compared two by two using V, the percentage of concordance: *V* = 2|*A* − *B*|/ (*A* + *B*) where A represents the parasite density reported by reader 1, B reader 2. If, V was < 30%, the parasite density was calculated as the geometric mean of the two values. If V was > 30%, the third reading was used to do the same calculation with A and B. Where A + C or B + C the value is less than 30%, the reading was validated.

### Mosquito membrane feeding assay

A direct mosquito feeding assay procedure (DMFA) previously described [36], was used to estimate drug effect on *P. falciparum* gametocyte infectivity and mosquito life history traits. From each blood sample, approximately 500 μl of blood in heparinized tubes was distributed to each of two membrane feeders and maintained at 37 ℃ by circulating water [36]. A mosquito colony of *An. gambiae* was used. The colony was established in 2008 from wild-caught gravid females collected in Soumousso (40 km southeast of Bobo-Dioulasso) and repeatedly replenished with F1 from wild-caught mosquito females collected in the same the village. Before the exposure to a blood meal, 3–5 days old female mosquitoes were kept without sucrose solution for 24 h. For each blood sample, two cups each containing 40 female mosquitoes were placed under the feeders to allow blood feeding through parafilm membranes for 30 min. Partially fed and unfed mosquitoes were discarded. Fed mosquitoes were kept in cages (30 × 30 × 30 cm) in the insectary with constant access to 5% glucose solution on cotton wool pads. Among 550 participants from whom blood could be collected, blood samples obtained from seven subjects could not be used for DMFA for logistics reasons (time between blood collection and exposure to mosquitoes above 4 h or lack of mosquitoes). On day 7 after membrane feeding, the midguts of mosquitoes of all surviving females were dissected and stained with 0.4% mercurochrome in phosphate buffered saline (PBS), pH 7.2. The presence and number of oocysts was recorded for each mosquito by microscopy. The success of mosquito infection was estimated by determining both oocyst prevalence (proportion of *P. falciparum*-infected mosquitoes) and density of infection (mean number of oocysts among infected mosquitoes).

### Mosquito life history traits

The effect of SPAQ and AZ on the blood-feeding rate and survival was investigated. Because the amount of blood ingested can influence the fecundity and survival of the mosquito, blood meal size was considered as a parameter of mosquito life trait. For this purpose, approximately 80 female mosquitoes (2 cups), in addition to the mosquitoes dedicated to transmission assays, were fed to estimate their life history traits using blood samples collected from 77 children in the three study groups. The fed mosquitoes were placed individually in 30-ml drosophila plastic tubes and kept in the insectary.

The blood-feeding rate of mosquitoes exposed to a blood meal was determined by calculating the ratio of fed mosquitoes compared to exposed mosquitoes. Mortality among fed mosquitoes was recorded every 8 h from the day of membrane blood feeding until death of all mosquitoes. Dead mosquitoes were removed from their individual tubes. Each tube was kept at + 4 ℃ before estimation of blood meal size. The size of the blood meal was measured by quantification of haematin, a product of digestion of haemoglobin and excreted in faeces of fed mosquitoes. The quantity of excreted haematin was estimated by adding 1 ml of 1% lithium carbonate (LiCO3) to individual tubes to elute faeces and the absorbance of the resulting solution was read at 387 nm in a Thermo Scientific Multiskan colorimeter. A LiCO3 solution was used as a blank. Absorbances were compared with a standard curve made with porcine serum haematin to obtain a relative measure of blood meal sizes [37].

### Statistical analysis

All statistical analyses were performed in R (version 3.4.0). Logistic regression by generalized linear mixed models (GLMM, binomial errors, logit link; lme4 package) was used to investigate the effect of treatment on: (i) asexual parasite prevalence; (ii) gametocyte prevalence; (iii) oocyst prevalence, (proportion of dissected mosquitoes with at least one oocyst in the midgut); and, (iv) mosquito blood-feeding rate. GLMM with negative binomial errors (lme4 package, glmer.nb function) [38] was used to test the effect of treatment on: (i) asexual parasitaemia; (ii) gametocytaemia in participants; and, (iii) oocyst density in mosquitoes. Because blood samples were not fully independent (some participants were sampled multiple times over time, i.e., pseudo-replication), participant identity was included as a random effect in all GLMMs. The effect of treatment on the proportion of infectious feeds was analysed using a binomial GLM. An ANOVA was used to explore the effect of treatment on mosquito blood meal size following a log transformation of haematin concentration. Finally, the effect of treatment on mosquito survivorship was analysed using a mixed effect Cox’s proportional hazard regression models (coxme package). Model simplification used stepwise removal of terms, followed by likelihood ratio tests (LRT). Term removals that significantly reduced explanatory power (p < 0.05) were retained in the minimal adequate model [39]. Multiple pairwise post-hoc tests were performed using the package ‘Multcomp’.

## Results

### Effect of treatment on the human parasite population

**Fig. 1:**
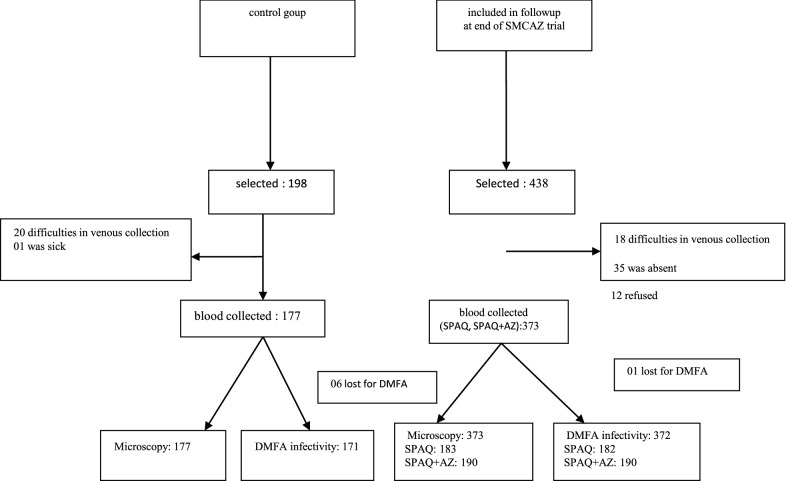
Summary of the transmission trial. The numbers represent the number of volunteers included at each step. DMFA: Direct membrane feeding assay

**Fig. 2:**
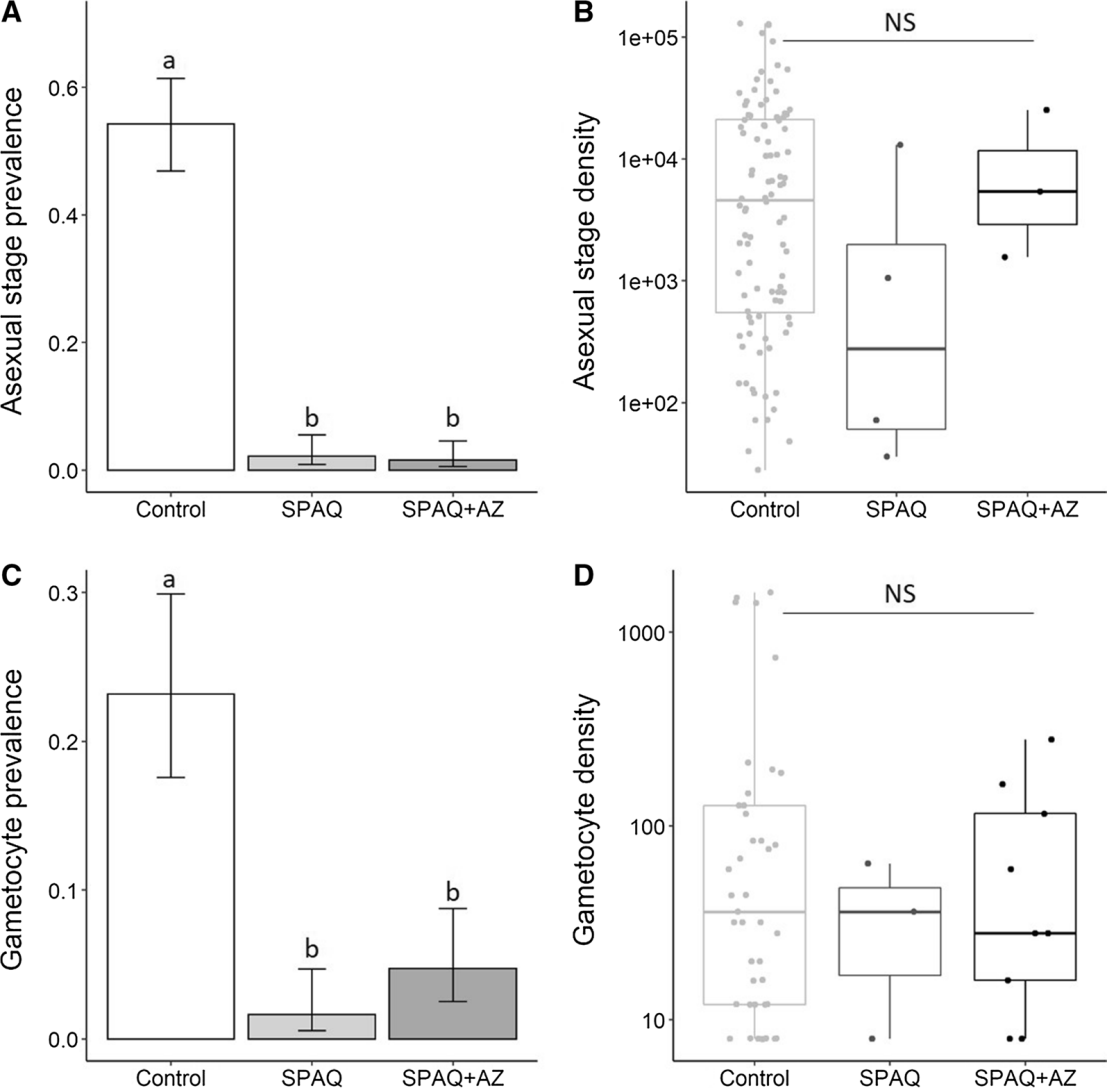
Effects of SPAQ and SPAQ + AZ on *Plasmodium falciparum* infection in humans. **A** The prevalence (± 95% CI) of asexual stage parasitaemia for each treatment and the control group. **B** The density of asexual stage parasites (number of parasites/µl of blood in infected blood collections) for each treatment and the control group. Each point represents a blood sample from *P. falciparum*-positive slides. The horizontal line represents the median value of parasite density for each of the three groups, and the upper and lower boundaries of the box indicate the 75th and 25th percentile, respectively. Note that the y-axis is on a log10 scale. **C** The prevalence (± 95% CI) of gametocyte for each treatment and the control group as measured by microscopy; **D** The density of gametocyte (number of gametocytes/µl of blood in gametocyte-positive blood collections) for each group. Note that the y-axis is on a log_10_ scale. The presence and number of asexual stages or gametocytes in samples was determined by microscopic observation. Different lowercase letters above the bars denote statistically significant differences based on multiple pair-wise post-hoc tests. NS: Not significant

A total of 550 blood collections were obtained from children aged 24–59 months. Among them, 373 had received chemoprevention (183 SPAQ and 190 SPAQ + AZ) (Fig. 1). The average time between drug administration and blood collection was similar in the two treatment groups, with 16.65 and 16.43 days for the SPAQ and SPAQ + AZ groups, respectively (W = 15,626, P = 0.35). The samples were not fully independent as some participants were tested more than once; two participants were sampled three times and 35 other participants were sampled twice. The proportion of children with slides positive for asexual stages of *Plasmodium sp* in the control group was 55.9% ± 7% (99/177). Among the 99 positives slides, 96 were positive for *P. falciparum* (97%), including 9 co-infections with *Plasmodium malariae*, 6 with *Plasmodium ovale* and 1 triple infection *P. falciparum*, *P. ovale* and *P. malariae*. There were only two mono-infections of *P. malariae* and one mono-infection of *P. ovale*. In the SPAQ treatment group, there were 4/183 (2.19%) slides positive for *P. falciparum* asexual stages. In the SPAQ + AZ treatment group 3/190 (1.58%) slides were positive. The treatments SPAQ and SPAQ + AZ significantly reduced the prevalence of asexual *P. falciparum* (LRT X^2^_2_ = 69, P < 0.0001, Fig. 2A).

There was no difference in *P. falciparum* asexual prevalence between SPAQ and SPAQ + AZ groups (multiple pairwise comparison: z = 0.4, P = 0.9). Among infected children in the control group, the mean *P. falciparum* asexual parasite density was 16,074 ± 2803 parasites/µl (range: 28–128,800). The four infected individuals of the SPAQ group had a mean asexual parasitaemia of 3548 ± 3167 parasites/µl (range: 36–13,022) and among the three positive children from the SPAQ + AZ group the mean density was 10,709 ± 7310 parasites/µl (range: 1560–25,160). Asexual parasite density was not significantly different among the three groups (LRT X^2^_2_ = 2.27, P = 0.32; Fig. 2B).

Chemoprevention with SPAQ or SPAQ + AZ treatments, significantly reduced the *P. falciparum* gametocyte prevalence (LRT X^2^_2_ = 54, P < 0.0001). There were 3/183 (1.6%) gametocyte-positive slides in the SPAQ group and 9/190 (4.7%) in the SPAQ + AZ group compared to a prevalence in the control group of 27% ± 6.5 (48/177). There was no difference in gametocyte prevalence between SPAQ and SPAQ + AZ groups (multiple pairwise comparison: z = 1.6, P = 0.2). The mean number of gametocytes/µl of blood was not influenced by the treatments (LRT X^2^_2_ = 0.73, P = 0.69; Fig. 2D).

### Effect of treatment on malaria transmission from humans to mosquitoes

**Fig. 3:**
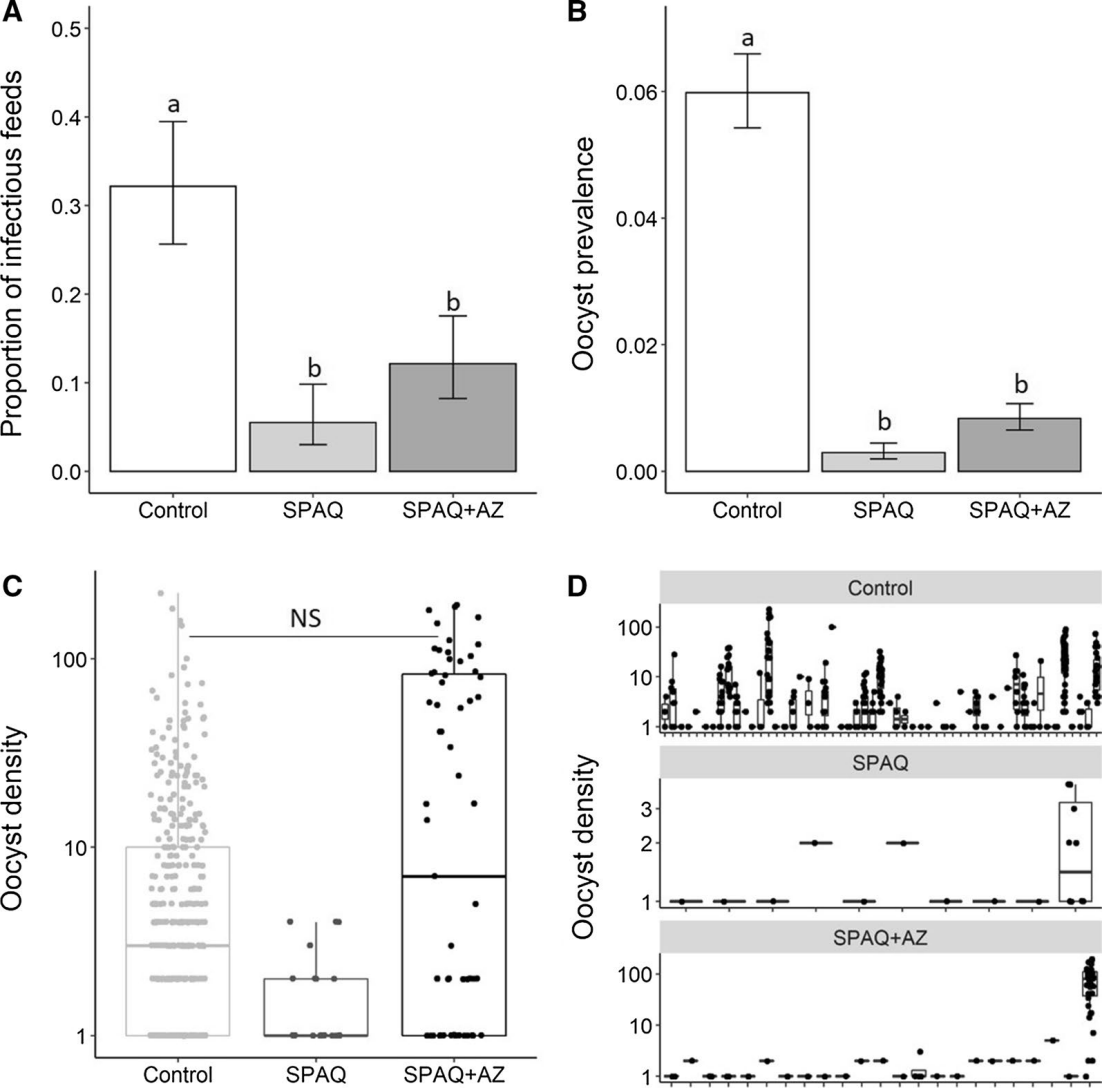
Effects of SPAQ and SPAQ + AZ on *Plasmodium falciparum* infectivity from humans to mosquitoes. **A** The proportion (± 95% CI) of feeds resulting in at least one successful mosquito infection (i.e., a minimum of one mosquito harbouring a minimum of one oocyst) for each treatment group. There were 171 feeds for the control, 182 for SPAQ and 190 for the SPAQ-AZ treatment and an average of 38.87 ± 0.48 (median = 41, range = 5–51) mosquitoes were dissected per feed (n total = 21,108 mosquitoes). **B** Oocyst prevalence (± 95% CI) defined as the total number of mosquitoes fed with blood drawn from each study group harbouring at least one oocyst in their midgut out of the number of dissected mosquitoes from this group. **C** Oocyst density defined as the number of oocysts observed in infected mosquitoes for each group. The horizontal line represents the median value of density for each of the three study groups, and the upper and lower boundaries of the box indicate the 75th and 25th percentile, respectively. **D** Oocyst density following infectious feeds on samples from the control group, SPAQ group and SPAQ + AZ group. Note that the y-axis on panels (C) and (D) is on a log_10_ scale. Different lowercase letters above the bars denote statistically significant differences based on multiple pair-wise post-hoc tests. NS: Not significant

A total of 21.108 mosquitoes, fed with one of 543 blood samples (171 from the control group, 182 from the SPAQ treatment group. and 190 from the SPAQ + AZ treatment group) through direct membrane feeding assays, were dissected seven days post feeding. Mosquitoes were successfully infected from a total of 88 blood samples (55 feeds from the control group, 10 feeds from the SPAQ treatment group and 23 from the SPAQ + AZ treatment group). The proportion of feeds giving a successful mosquito infection varied significantly among the groups (LRT X^2^_2_ = 61, P < 0.0001; Fig. 3A). Thirty-two per cent (55/171) of the blood feeds originating from the control group resulted in successful mosquito infection. The proportion of infectious feeds from the SPAQ + AZ group (23/190) was twice as large as that from the SPAQ group (10/182) (LRT X^2^_1_ = 5.2, P = 0.02, multiple pair wise comparison: z = 2.2, P = 0.07, Fig. 3A), suggesting a significant effect of AZ on gametocyte infectivity.

The prevalence of oocysts (i.e., the number of mosquitoes presenting at least one oocyst in the midgut out of the total number of dissected mosquitoes) was higher in the control group than in the two treatment groups (*X*^*2*^_2_ = 22.8, P < 0.001, Fig. 3B). Following 55 infectious feeds on sample from the control group, including 25 from gametocyte-positive blood samples (19 for *P. falciparum*, 1 for *P. ovale*, 1 for *P. malariae*, 3 mixed infections with *P. falciparum* and *P. malariae* and 1 with *P. falciparum*, *P. ovale* and *P. malariae*), 380 out of 6,349 dissected mosquitoes (6%), were infected (Fig. 3B). In the SPAQ group, 21 out of 7,207 mosquitoes (0.29%) were infected from 10 infectious feeds, including one from a *P. falciparum* gametocyte-positive blood collection (Fig. 3B). In the SPAQ + AZ group, 63 out of 7,552 mosquitoes (0.8%) were infected from 23 infectious feeds, including 3 from *P. falciparum* gametocyte-positive blood samples (Fig. 3B). There was no difference in oocyst prevalence between SPAQ and SPAQ + AZ groups (multiple pair wise comparison: z = 1.3, P = 0.4).

Although mosquitoes infected from blood samples obtained from the SPAQ + AZ group harboured relatively more oocysts in their midguts (mean ± se density of 43 ± 7) compared to mosquitoes infected from samples obtained from the control (10.9 ± 1.2) or SPAQ (1.8 ± 0.3) groups, the difference was not statistically significant (LRT *X*^*2*^_2_ = 4.4, P = 0.11, Fig. 3C). This is because more than half of the mosquito infections in the SPAQ + AZ group (34/63) came from a single gametocyte carrier. Figure 3D shows the oocyst density produced by each infectious feed (n = 55 for the control group, 10 for SPAQ and 23 for SPAQ + AZ). Most infectious feeds from the SPAQ or SPAQ + AZ group resulted in single infected mosquitoes harbouring very few oocysts (Fig. 3D).

### Effect of treatment on mosquito blood-feeding success, mosquito blood-meal size and on mosquito survival

**Fig. 4:**
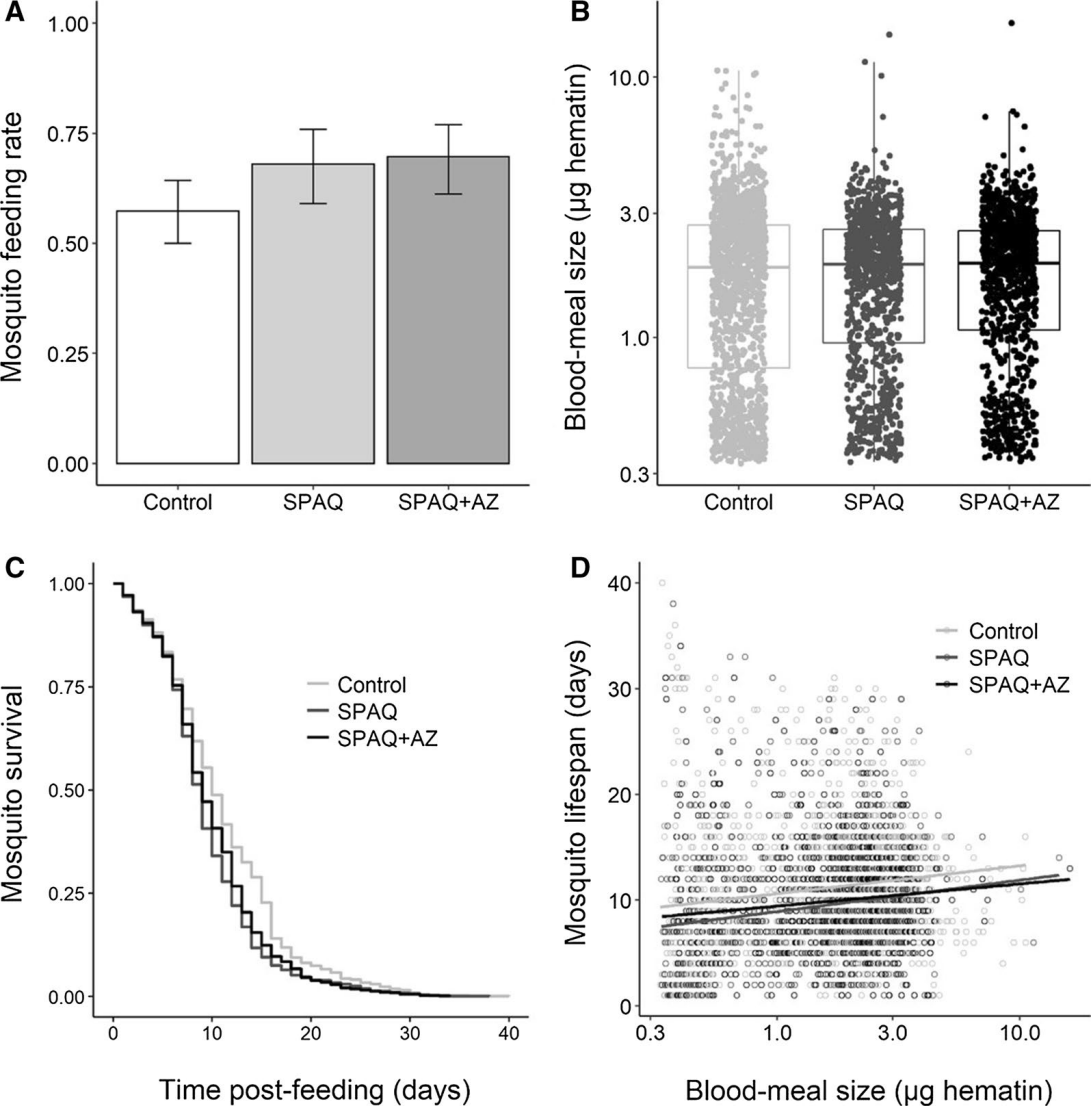
Effect of SPAQ and SPAQ + AZ on mosquito life history traits**. A** Mosquito blood-feeding rate, expressed as the proportion (+ 95% CI) of engorged mosquitoes out of the total number exposed to the blood meal; **B** Mosquito blood-meal size, expressed as the quantity of haematin in µg excreted by individual mosquitoes. Note that the y-axis is on a log_10_ scale. **C** The daily survival of mosquitoes individually kept in drosophila tubes. **D** The relationship between mosquito lifespan and blood meal size. Note that the x-axis is on a log_10_ scale

To investigate the effect of treatment on mosquito blood-feeding success, the proportion of fully engorged females was recorded for 34 feeds on blood samples from the control group (2547 mosquitoes), 19 feeds from the SPAQ group (1,491 mosquitoes) or 21 feeds from the SPAQ + AZ group (1647 mosquitoes). A marginally significant effect of treatment on mosquito feeding rate was observed (LRT*X*^*2*^_2_ = 6.0, P = 0.048, Fig. 4A), with slightly lower feeding success with blood samples from the control group.

To investigate the effect of treatment on mosquito blood-meal size and mosquito survival, fully fed females from 35 feeds on blood samples from the controls group (1440 mosquitoes), 21 feeds from the SPAQ group (947 mosquitoes) or 21 feeds from the SPAQ + AZ group (1,055 mosquitoes) were kept individually in drosophila plastic tubes. The size of the blood meal was measured following the haematin dosage procedure. There was no effect of SPAQ or SPAQ + AZ on mosquito blood meal size (LRT X^2^_2_ = 3.0, P = 0.12, Fig. 4B).

The daily mortality of individual female mosquitoes was recorded. There was a slight but statistically significant negative effect of SPAQ and SPAQ + AZ on mosquito survival compared to mosquitoes fed with blood samples from the control group (median longevity of mosquitoes 10, 9 and 9 days for the control, SPAQ and SPAQ + AZ groups, respectively, LRTX^2^_2_ = 330, P < 0.0001, Fig. 4C, Table 1, multiple pair-wise comparisons, control-SPAQ: z = 3.338, P = 0.002, control-SPAQ + AZ: z = 2.462, P = 0.036, SPAQ-SPAQ + AZ: z = 0.8, P = 0.7).

**Table 1:** Risk of mortality (hazard ratio) along with the standard error, z and P-value for each treatment group relative to the control group

Treatment	Hazard ratio (se)	z	P-value
SPAQ	1.486 (0.119)	3.34	0.0008
SPAQ + AZ	1.333 (0.117)	2.46	0.014

There was a significant positive relationship between mosquito lifespan and blood meal size (LRT *X*^*2*^_1_ = 142, P < 0.001, Fig. 4D), with females that took large blood meals living longer than those that took smaller blood meals. This relationship was true for all three groups (treatment by blood meal size interaction: LRT *X*^*2*^_2_ = 1.5, P = 0.17, Fig. 4D).

## Discussion

The present study investigated the effect of SMC with or without the addition of AZ on transmission of malaria. A significant reduction in malaria prevalence was found among blood samples obtained from treated children (SPAQ or SPAQ + AZ) compared to samples from the control group but no significant difference was seen between the SPAQ and SPAQ + AZ groups. This finding indicates that addition of AZ does not influence the malaria prevalence in the context of SMC as previously observed [4]. Consistently, the prevalence of gametocytes was significantly lower in children treated with SPAQ or SPAQ + AZ than in the controls but there was no difference between SPAQ and SPAQ + AZ groups. These findings indicate that SMC with SPAQ may have an effect on the overall transmission of malaria, especially if SMC was extended to older children, but that addition of AZ will not enhance this effect.

The proportion of mosquitoes infected from blood samples obtained from children in the control group was significantly higher than that in mosquitoes fed on blood from children in the SPAQ or SPAQ + AZ groups. The low rate of infectivity of post-treatment patients may be due to the cumulative effects of the treatment; that the sporontocidal activity of SP is known to affect parasite development prior to the stage of mature oocyst when SP is ingested together with gametocytes during the mosquito blood meal [12, 36] and the rapid action of AQ on asexual forms that limits the presence and number of asexual parasites that engage in the gametocytogenesis [15, 40, 41].

A higher proportion of mosquitoes fed on blood samples from children in the SPAQ + AZ group were infectious compared to feeds on samples from children in the SPAQ group suggesting that mosquitoes could be more prone to become infected with *Plasmodium* when feeding on blood containing AZ. Two hypotheses may explain the present result. Firstly, despite the relatively short half-life of AZ (44 h) [42], AZ remains detectable for more than 20 days, especially in white blood cells [43] and AZ ingested by mosquitoes during blood feeding may increase mosquito permissiveness to malaria infection by affecting their midgut microbiota and subsequently impact on microbe-parasite interactions, as previously observed with other antibiotics [19, 27]. However, this contrasts with previous findings, which showed that AZ added to an infectious blood meal reduced the parasite load in mosquitoes [28]. Dose-dependent effects of antibiotics on malaria transmission could account for these different findings. Secondly, mechanisms occurring in the human host could explain the influence of AZ on infectivity of gametocytes to mosquitoes. Because gametocyte density in infected individuals is often too low to be detected by standard light microscopy [44, 45], it cannot be excluded that the AZ treatment increases the prevalence of gametocyte carriage and subsequently increases transmission despite low densities of sexual parasites. Molecular detection and quantification of gametocytes may help understanding the effect of AZ administration on malaria transmission.

A marginal effect of the treatment with SPAQ or SPAQ + AZ on the mosquito feeding rate was observed. Mosquitoes seemed to be more prone to complete a blood meal when blood was collected from children who had received treatment compared to controls. This suggests that SPAQ or SPAQ + AZ influence mosquitoes feeding behaviour, increasing appetite. This may be due to the presence of drug metabolites still present in the blood. Indeed, it has been demonstrated that several chemical families may influence the attraction and feeding behaviour of the vector mosquitoes [46].

Transmission of *Plasmodium* is closely dependent on the ability of the mosquito to harbour the parasite and to survive long enough for them to develop. The probability of mosquito survival was significantly lower in the treated groups (SPAQ, SPAQ + AZ) than in the control group, with a median longevity one day shorter, suggesting that the drugs reduced mosquito survival in accordance with previous observations [17, 28]. Although the observed reduction of survival is moderate in this study, probably due to low concentrations of drugs in blood collected 14 to 21 days post treatment, the reduction of mosquito life span could significantly disturb the *Plasmodium* sporogonic cycle. Indeed, a decreased mosquito survival rate strongly affects vectorial capacity, by decreasing the likelihood of infected mosquitoes surviving beyond the parasite extrinsic incubation period (time required between being ingested and becoming infective to humans), and therefore the probability of transmitting the disease to new hosts [47].

The moderate lethal effect of the molecules on the mosquito feeding rate and on mosquito longevity would have been more significant if the blood had been drawn in at an interval of time less than 14 days when the concentration of the molecules is higher in the blood. Therefore, subsequent studies could be carried out by taking blood at different times points after the treatment to determine the relation between the time of chemoprevention administration and it effect on mosquito fitness and parasite transmission.

## Conclusion

This study, conducted in Burkina Faso, showed that SMC greatly reduces the infectious parasite reservoir, decreases gametocyte carriage and infectivity to mosquitoes. The finding indicates that the addition of AZ to SPAQ prophylaxis slightly influences human to mosquito malaria transmission, with an increased proportion of infectious mosquito feeds 2 to 3 weeks after the start of treatment in children with AZ or SPAQ. Despite the fact that this effect was marginal and could be due to chance, this is a potentially undesirable effect of adding AZ to SMC and does not support mass administration of this antibiotic in the context of malaria transmission.

The result also showed that SMC using SPAQ, with or without AZ, affects the mosquito’s life traits with a slightly enhanced blood feeding success and a decrease in mosquito survival rate. The importance of mosquito longevity in malaria transmission suggests that, between these two effects on mosquito’s life traits, the effect on survival may be the most important in terms of vectorial capacity. This suggests that in addition to the prophylactic effect in humans and the reduction of transmission to mosquitoes, SMC may also affect mosquito longevity in a way that confirms its value in the fight against malaria.

The findings show a strong benefit of SMC in reducing human to mosquito transmission. However, although the effect was only moderate, the results show that the addition of AZ to SPAQ in SMC might favour malaria transmission and does not support mass administration of this drug in the context of malaria control.

## Data Availability

The data used in this article is available to readers.

## Abbreviations

IPTp: Intermittent preventive treatment during pregnancy
SMC: Seasonal malaria chemoprevention
SP: Sulfadoxine pyrimethamine
AQ: Amodiaquine
AZ: Azithromycin
DMFA: Direct membrane feeding assay
PBS: Phosphate buffered saline
LiCO3: Lithium carbonate
GLMM: Generalized linear mixed models
LRT: Likelihood ratio tests
